# KIF2C: a novel link between Wnt/β-catenin and mTORC1 signaling in the pathogenesis of hepatocellular carcinoma

**DOI:** 10.1007/s13238-020-00766-y

**Published:** 2020-08-03

**Authors:** Shi Wei, Miaomiao Dai, Chi Zhang, Kai Teng, Fengwei Wang, Hongbo Li, Weipeng Sun, Zihao Feng, Tiebang Kang, Xinyuan Guan, Ruihua Xu, Muyan Cai, Dan Xie

**Affiliations:** 1 State Key Laboratory of Oncology in South China, Collaborative Innovation Center for Cancer Medicine, Sun Yat-sen University Cancer Center, Guangzhou, 510060 China; 2 Department of Radiation Oncology, Shandong Cancer Hospital and Institute, Shandong First Medical University and Shandong Academy of Medical Sciences, Jinan, 250200 China; 3 Department of Musculoskeletal Oncology, the First Affiliated Hospital, Sun Yat-sen University, Guangzhou, 510080 China; 4 Department of Anorectal Surgery, the First Affiliated Hospital of Zhengzhou University, Zhengzhou, 510370 China; 5 Department of Urology, the First Affiliated Hospital, Sun Yat-sen University, Guangzhou, 510080 China; 6 Department of Clinical Oncology, Li Ka Shing Faculty of Medicine, The University of Hong Kong, Pokfulam, Hong Kong SAR China; 7 Department of Pathology, Sun Yat-sen University Cancer Center, Guangzhou, 510060 China

**Keywords:** KIF2C, HCC, TBC1D7, mTORC1 signaling, Wnt/β-catenin signaling

## Abstract

Hepatocellular carcinoma (HCC) is the most common primary liver malignancy and is the fourth-leading cause of cancer-related deaths worldwide. HCC is refractory to many standard cancer treatments and the prognosis is often poor, highlighting a pressing need to identify biomarkers of aggressiveness and potential targets for future treatments. Kinesin family member 2C (KIF2C) is reported to be highly expressed in several human tumors. Nevertheless, the molecular mechanisms underlying the role of KIF2C in tumor development and progression have not been investigated. In this study, we found that KIF2C expression was significantly upregulated in HCC, and that KIF2C up-regulation was associated with a poor prognosis. Utilizing both gain and loss of function assays, we showed that KIF2C promoted HCC cell proliferation, migration, invasion, and metastasis both *in vitro* and *in vivo*. Mechanistically, we identified TBC1D7 as a binding partner of KIF2C, and this interaction disrupts the formation of the TSC complex, resulting in the enhancement of mammalian target of rapamycin complex1 (mTORC1) signal transduction. Additionally, we found that KIF2C is a direct target of the Wnt/β-catenin pathway, and acts as a key factor in mediating the crosstalk between Wnt/β-catenin and mTORC1 signaling. Thus, the results of our study establish a link between Wnt/β-catenin and mTORC1 signaling, which highlights the potential of KIF2C as a therapeutic target for the treatment of HCC.

**Electronic supplementary material:**

The online version of this article (10.1007/s13238-020-00766-y) contains supplementary material, which is available to authorized users.

## Introduction

Hepatocellular carcinoma (HCC) is the most common type of primary liver cancer and is currently ranked as the fourth-leading cause of cancer-related deaths worldwide (Bray et al., 2018). HCC has a high prevalence in Southeast Asia and Africa, and its incidence and mortality have been steadily increasing in Europe and America (El-Serag and Mason, 1999; Ince and Wands, 1999). HCC is a complex and highly heterogeneous cancer that has been associated with various genomic alterations and aberrant activation of different molecular pathways (Tanaka and Arii, 2012). Despite the plethora of studies on the pathogenesis of HCC in recent decades, the underlying molecular mechanisms are still not fully understood. The prognosis of HCC patients remains dismal, which is in part due to its insidious onset, resulting in a delayed diagnosis, its aggressive malignancy, and its high propensity for metastasis. Many of the standard treatment options for cancer patients, including chemotherapy drugs and radiotherapy, are not highly effective in the treatment of HCC. In the majority of cases, the only curative treatment options include surgical resection and liver transplantation, both of which share a high tumor recurrence rate (Njei et al., 2015). Thus, it is of paramount importance to further elucidate the underlying pathogenesis of HCC in order to identify novel therapeutic targets.

The mammalian target of rapamycin (mTOR) is a serine/threonine kinase that regulates cell growth, proliferation, and metabolism (Saxton and Sabatini, 2017). mTOR was identified and characterized as the catalytic core in two distinct protein complexes, known as mTOR complex 1 (mTORC1) and mTOR complex 2 (mTORC2). The activity of mTORC1 is negatively regulated by the TSC complex, which is comprised of tuberous sclerosis complex 1 (TSC1), tuberous sclerosis complex 2 (TSC2), and Tre2-Bub2-Cdc16 (TBC) 1 domain family, member 7 (TBC1D7) (Dibble et al., 2012; Saxton and Sabatini, 2017). The TSC complex functions as a GTPase activating protein (GAP) of Ras homolog enriched in brain (Rheb) (Zhang et al., 2003), and is an essential activator of mTORC1, which is dependent upon its GTP-bound state (Saucedo et al., 2003). Upon activation, mTORC1 subsequently phosphorylates its substrates, Ribosomal S6 kinase (S6K) and eukaryote initiation factor 4E binding protein 1 (4EBP1). These two substrates are key factors in controlling protein translation and cell growth (Harris and Lawrence, 2003; Jacinto and Hall, 2003). The deregulation of mTORC1 signaling has been implicated in the progression of various human cancers. Recently, mTORC1 signaling has been shown to be a major tumor-initiating pathway in HCC (Bhat et al., 2013). However, the detailed regulation of mTORC1 signaling in HCC progression has yet to be elucidated.

Kinesin family member 2C (KIF2C), a member of the kinesin 13 family, is an M-kinesin and contains a motor domain in the middle of its amino acid sequence (Hirokawa et al., 2009; Ritter et al., 2016). KIF2C has been shown to be essential for mitosis through its participation in spindle assembly, chromosome congression and segregation, and kinetochore-microtubule attachment (Moore et al., 2005; Lee et al., 2008; Honnappa et al., 2009; Tanenbaum et al., 2011). Additionally, KIF2C participates in cytoskeletal remodeling during migration and invasion (Braun et al., 2014; Eichenlaub-Ritter, 2015; Ritter et al., 2015). KIF2C has also been identified as a putative oncogene that is highly expressed in breast cancer, gastric cancer, colorectal cancer, glioma, and non-small cell lung cancer (Nakamura et al., 2007; Ishikawa et al., 2008; Shimo et al., 2008; Bie et al., 2012; Gan et al., 2019). KIF2C has been reported to be involved in the proliferation and migration of gastric cancer cells and non-small cell lung cancer cells, however, the precise role and mechanism remain unclear (Nakamura et al., 2007; Gan et al., 2019). Despite the limited functional studies regarding KIF2C in carcinogenesis, there is enough evidence to suggest that KIF2C may play a role in HCC progression.

In this study, we report that the expression level of KIF2C is increased in HCC tissues, and that higher expression level is closely correlated with being an aggressive carcinoma and having a poor prognosis. Both *in vitro* and *in vivo* experiments demonstrate that KIF2C plays a strong oncogenic role in the progression of HCC. Mechanistic studies reveal that KIF2C strengthens mTORC1 signaling by interacting with TBC1D7 to disturb the formation of the TSC complex. Furthermore, we found that KIF2C expression is directly upregulated by Wnt/β-catenin signaling, and the upregulation of KIF2C mediates the activation of the mTORC1 pathway. Our findings provide functional and mechanistic links among KIF2C, Wnt/β-catenin and mTORC1 signaling in the aggressive nature of HCC.

## Results

### KIF2C is upregulated in HCC and correlated with a poor grognosis

**Figure 1:**
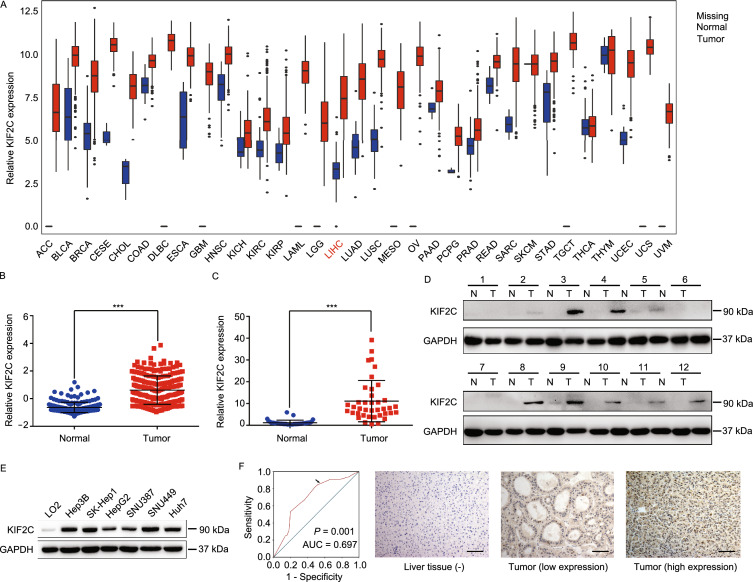

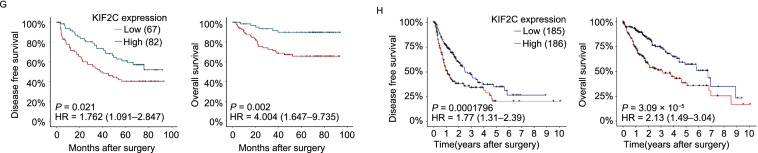
**KIF2C upregulation is frequently observed in HCC and correlates with a poor prognosis**. (A) Expression of KIF2C mRNA in different cancers (tumor vs. corresponding normal tissues) was analyzed based on the data from The Cancer Genome Atlas project. ACC: adrenocortical carcinoma; BLCA: bladder urothelial carcinoma; BRCA: breast invasive carcinoma; CESE: cervical squamous cell carcinoma and endocervical adenocarcinoma; CHOL: cholangiocarcinoma; COAD: colon adenocarcinoma; DLBC: lymphoid neoplasm diffuse large B-cell lymphoma; ESCA: esophageal carcinoma; GBM: glioblastoma multiforme; HNSC: head and neck squamous cell carcinoma; KICH: kidney chromophobe; KIRC: kidney renal clear cell carcinoma; KIRP: kidney renal papillary cell carcinoma; LAML: acute myeloid leukemia; LGG: brain lower grade glioma; LIHC: liver hepatocellular carcinoma; LUAD: lung adenocarcinoma; LUSC: lung squamous cell carcinoma; MESO: mesothelioma; OV: ovarian serous cystadenocarcinoma; PAAD: pancreatic adenocarcinoma; PCPG: pheochromocytoma and paraganglioma; PRAD: prostate adenocarcinoma; READ: rectum adenocarcinoma; SARC: sarcoma; SKCM: skin cutaneous melanoma; STAD: stomach adenocarcinoma; TGCT: testicular germ cell tumors; THCA: thyroid carcinoma; THYM: thymoma; UCEC: uterine corpus endometrial carcinoma; UCS: uterine carcinosarcoma; UVM: uveal melanoma. (B) KIF2C mRNA expression from an OncoMine microarray data set, including 225 HCC and 220 normal liver tissues. Data are presented as the mean ± SD, ***P < 0.001, Student’s *t*-test. (C) qRT-PCR analysis of KIF2C mRNA expression in 39 paired HCC and adjacent non-tumor tissues. Data are presented as the mean ± SD of three independent experiments, ***P < 0.001, Student’s *t*-test. (D) Western blot analysis of the KIF2C protein expression levels in 12 pairs of HCC and adjacent normal tissues. (E) Expression of KIF2C in immortalized hepatocytes and HCC cell lines examined by Western blot. (F) Receiver operating characteristic curve analysis was performed to determine the cut-off score for the high expression of KIF2C (left panel). Representative immunohistochemical images show the expression pattern of KIF2C in the HCC and adjacent liver tissues (right panel). Scale bars, 100 μm. (G) Kaplan-Meier survival analysis of disease-free survival (DFS) or overall survival (OS) according to KIF2C expression in 149 patients with HCC (log-rank test). (H) Kaplan-Meier survival analysis of DFS or OS according to KIF2C expression from TCGA database in patients with HCC (log-rank test)

Analysis of the data in The Cancer Genome Atlas (TCGA) database indicated that KIF2C expression was increased in almost all cancer types, including HCC (Figs. 1A and S1A). The upregulation of KIF2C in HCC was shown to be the highest among the kinesin family proteins (Fig. S1B). Consistent with these results, the data in Oncomine, from a transcriptome study of 225 primary HCC and 220 normal liver tissues, showed that KIF2C was significantly overexpressed in HCC compared to normal liver tissues (Fig. 1B) (Roessler et al., 2010). We verified these findings using qRT-PCR and Western blot analysis to examine the RNA and protein levels of KIF2C in HCC and the adjacent normal tissue samples, the results of which were in concordance with what we found in the online databases (Fig. 1C and 1D). Additionally, the protein expression of KIF2C was found to be elevated in all of the HCC cell lines in comparison to LO2, a normal human hepatic cell line (Fig. 1E).

Next, we performed an immunohistochemistry (IHC) analysis to further evaluate the expression status of KIF2C in HCC and the adjacent normal liver tissues. According to the ROC curve, we confirmed that KIF2C protein expression was upregulated in HCC tissues (Fig. 1F). Immunohistochemical staining of KIF2C in representative samples of HCC and liver tissues is shown in Fig. 1F. A correlation analysis showed that higher expression levels of KIF2C were closely associated with tumor differentiation and recurrence (*P* < 0.05, Table 1). Furthermore, a Kaplan-Meier analysis showed that patients with higher KIF2C protein levels had a lower rate of disease-free survival as well as a lower rate of overall survival (Fig. 1G). This finding was confirmed using the data from TCGA cohort (Fig. 1H). Multivariate Cox proportional hazard regression analysis further demonstrated that high expression levels of KIF2C represented an independent prognostic factor for the overall survival of HCC patients (HR 4.168, 95% CI 1.712–10.147, *P* = 0.002, Table 2).

**Table 1:** Correlation of KIF2C expression with patients’ clinicopathological features in primary hepatocellular carcinomas

Variable	KIF2C protein			
	All cases	Low expression	High expression	*P* value^*^
Age (years)				0.387
≤50	77	32 (41.6%)	45 (58.4%)	
>50	72	35 (48.6%)	37 (51.4%)	
Sex				0.332
Male	20	11 (55.0%)	9 (45.0%)	
Female	129	56 (43.4%)	73 (56.6%)	
HbsAg				0.469
Positive	23	12 (52.2%)	11 (47.8%)	
Negative	125	55 (44.0%)	70 (56.0%)	
AFP (ng/mL)^†^				0.429
≤20	60	25 (41.7%)	35 (58.3%)	
>20	87	42 (48.3%)	45 (51.7%)	
Liver cirrhosis				0.427
Yes	122	53 (43.4%)	69 (56.6%)	
No	27	14 (51.9%)	13 (48.1%)	
Tumor size (cm)				0.243
≤5	90	37 (41.1%)	53 (58.9%)	
>5	59	30 (50.8%)	29 (49.2%)	
Tumor multiplicity				0.220
Single	113	54 (47.8%)	59 (52.2%)	
Multiple	36	13 (36.1%)	23 (63.9%)	
Differentiation				0.016
Well	98	51 (52.0%)	47 (48.0%)	
Moderate-Poor	51	16 (31.4%)	35 (68.6%)	
Stage				0.570
I	74	35 (47.3%)	39 (52.7%)	
II-III	75	32 (42.7%)	43 (57.3%)	
Vascular invasion				0.895
Absent	97	44 (45.4%)	53 (54.6%)	
Present	52	23 (44.2%)	29 (55.8%)	
Relapse				0.025
Absent	76	41 (53.9%)	35 (46.1%)	
Present	73	26 (35.6%)	47 (64.4%)	

^*^Chi-square test; ^†^Preoperative serum AFP was not measured in two patients; HBV, hepatitis B virus; HCV, hepatitis B virus; AFP, alpha-fetoprotein

**Table 2:** Univariate and multivariate analysis of different prognostic features in 149 patients with hepatocellular carcinoma

Variable	Univariate analysis*			Multivariate analysis^†^	
	All cases	Mean survival (months)	*P* value	HR (95% CI)	*P* value
Age (years)			0.924		
≤50	77	75.9			
>50	72	76.8			
Sex			0.678		
Male	20	75.5			
Female	129	76.0			
HbsAg			0.270		
Positive	125	77.9			
Negative	23	69.1			
AFP (ng/mL)			0.182		
≤20	60	80.9			
>20	87	74.0			
Liver cirrhosis			0.047		
Yes	122	73.9			
No	27	87.0			
Tumor size (cm)			0.019		
≤5	90	80.5			
>5	59	65.3			
Tumor multiplicity			0.388		
Single	113	77.7			
Multiple	36	72.4			
Differentiation			0.146		
Well	98	79.5			
Moderate-Poor	51	70.3			
Stage			0.001		
I	74	85.0			
II-III	75	66.9			
Vascular invasion			<0.0001		<0.0001
Absent	97	84.2		1.0	
Present	52	56.9		4.255 (2.073–8.734)	
KIF2C expression			0.001		0.002
Low	67	86.6		1.0	
High	82	68.3		4.168 (1.712–0.147)	

*Log-rank test; ^†^Cox regression model; HR indicates hazards ratio; CI indicates confidence interval.

### KIF2C increases HCC cell proliferation both *in vitro* and *in vivo*

To clarify the role of KIF2C in HCC, we established cell lines using a lentiviral system to either knockdown or overexpress the KIF2C protein. We knocked down KIF2C in its high-expressing HCC cell lines SK-Hep1 and SNU449 using small hairpin RNA (shRNA), and overexpressed KIF2C in its low-expressing HepG2 and SNU387 cells, and the efficiencies of KIF2C silencing and overexpression were confirmed by Western blot (Fig. S2A and S2B).

**Figure 2:**
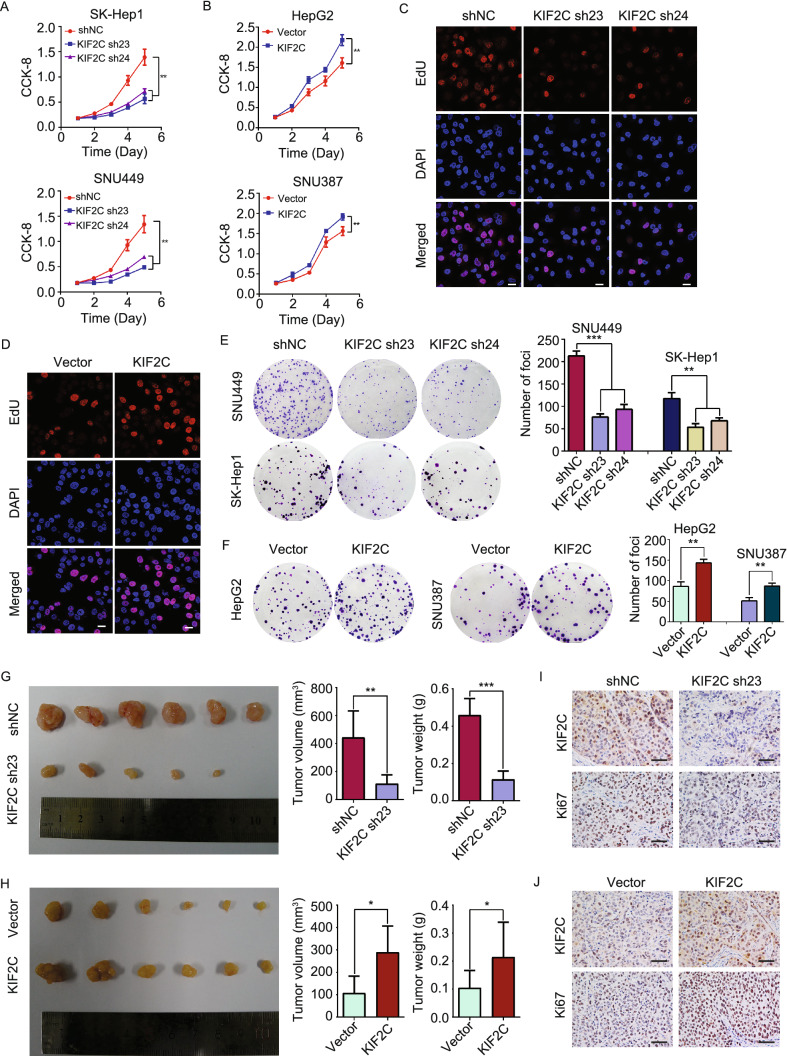
**KIF2C promotes HCC cell proliferation and tumor growth**. (A and B) Cell Counting Kit-8 (CCK-8) assay shows that the knockdown of KIF2C inhibits HCC cell proliferation (A), whereas KIF2C overexpression enhances HCC cell proliferation (B). (C and D) The influence of KIF2C silencing (C) and ectopic expression (D) on HCC cell proliferation determined by EdU staining assay in SK-Hep1 and HepG2 cells, respectively. Scale bars, 20 μm. (E and F) Colony formation images of KIF2C-knockdown (E) and KIF2C-overexpression (F) HCC cells. The colonies were captured and counted. Data are presented as the mean ± SD of three independent experiments, **P < 0.01, ***P < 0.001, Student’s *t*-test. (G and H) The growth of HCC subcutaneous tumors is suppressed by KIF2C silencing (G) while is enhanced by KIF2C overexpression (H). Subcutaneous tumors were measured by volume and weight. *P < 0.05, **P < 0.01, ***P < 0.001, Student’s *t*-test. (I and J) IHC analysis comparing the expression levels of KIF2C and the proliferation marker Ki-67 in the indicated subcutaneous tumors. Scale bars, 50 μm

Using the established cell lines, we were then able to assess the function of KIF2C in HCC cell growth and proliferation. As shown in Fig. 2A, the knockdown of KIF2C inhibited the proliferation of HCC cells, whereas KIF2C overexpression in the HepG2 and SNU387 cells was observed to have the opposite effect (Fig. 2B). Consistent with these results, the percentage of EdU-positive cells was decreased in KIF2C-depleted cells whereas it was increased in KIF2C-overexpressing cells (Figs. 2C, 2D, S2C and S2D). In accordance with these findings, silencing KIF2C abolished the colony formation of HCC cells, while ectopic expression of KIF2C enhanced the colony formation (Fig. 2E and 2F).

To further investigate whether KIF2C can also promote HCC cell proliferation *in vivo*, we established a xenograft tumor model by subcutaneously injecting the indicated HCC cells into nude mice. After four weeks, the mice were euthanized and the size and weight of the xenograft tumors were measured. The results showed that the average volume and weight of the tumors derived from the SK-Hep1 cell line with stable KIF2C knockdown were markedly lower than those from the sh-control cells (Fig. 2G). However, in the mice inoculated with the HepG2-KIF2C cells, the tumors were significantly larger and heavier than those in the control group (Fig. 2H). IHC analysis examining the expression of the proliferation marker Ki-67 showed that this marker was lower in the KIF2C-depleted SK-Hep1 xenografts compared to its expression in the SK-Hep1 control group, whereas it was higher in the HepG2-KIF2C xenografts in comparison to the control group (Fig. 2I and 2J). These results suggest that KIF2C has an oncogenic role in HCC and could enhance HCC proliferation both *in vitro* and in *vivo*.

### KIF2C promotes HCC cell migration, invasion, and metastasis

**Figure 3:**
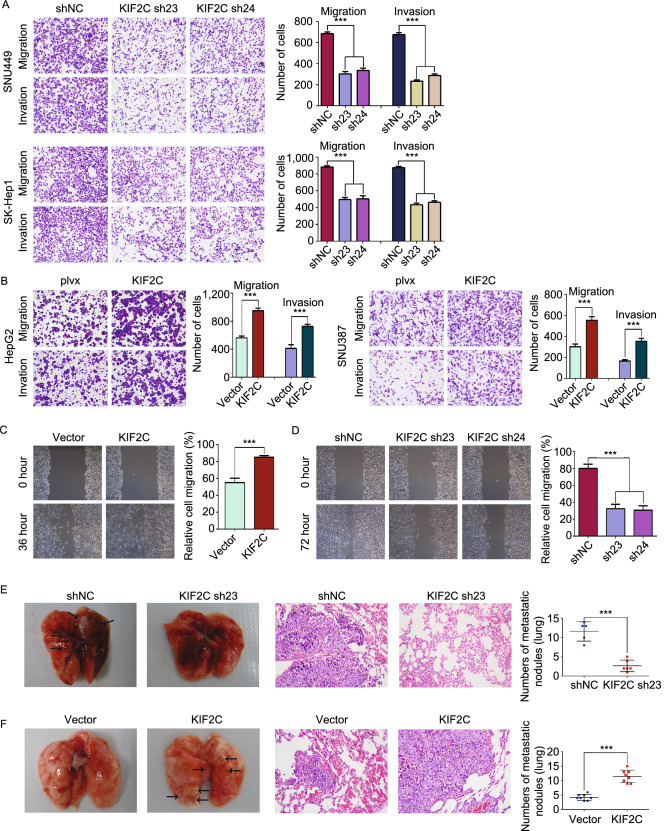
**KIF2C promotes HCC migration and invasion*in vitro*and*in vivo***. (A) Transwell migration and Matrigel invasion assays demonstrating that knockdown of KIF2C decreases the migratory and invasive abilities of HCC cells. The migrated and invaded cells were counted. Data are presented as the mean ± SD of three independent experiments, ***P < 0.001, Student’s *t*-test. (B) Transwell migration and Matrigel invasion assays showing that overexpression of KIF2C promotes the migratory and invasive abilities of HCC cells. Data are presented as the mean ± SD of three independent experiments, ***P < 0.001, Student’s *t*-test. (C) Wound healing assay revealing that KIF2C overexpression promotes HepG2 cell migration. Data are presented as the mean ± SD of three independent experiments, ***P < 0.001, Student’s *t*-test. (D) Wound healing assay revealing that silencing of KIF2C inhibits SK-Hep1 cell migration. Data are presented as the mean ± SD of three independent experiments, ***P < 0.001, Student’s *t*-test. (E) Lung metastasis model was established to test the effect of KIF2C silencing on tumor metastasis by tail vein injection of the indicated cells. Representative images of lungs and H&E staining of pulmonary metastatic tumors are shown. The tumor nodules in H&E staining slices were counted. Data are presented as the mean ± SD, ***P < 0.001, Student’s *t*-test. (F) Ectopic expression of KIF2C enhances HCC lung metastasis in nude mice. Representative images of lungs and H&E staining of pulmonary metastatic tumors are shown. The tumor nodules in H&E staining slices were counted. Data are presented as the mean ± SD, ***P < 0.001, Student’s *t*-test

We then investigated whether KIF2C affects HCC cell migration and invasion. Transwell migration and Matrigel invasion assays demonstrated that silencing KIF2C expression decreased the migration and invasion of SK-Hep1 and SNU449 cells (Fig. 3A). However, the migratory and invasive abilities of HepG2 and SNU387 cells were increased by the overexpression of KIF2C (Fig. 3B). In accordance with these findings, KIF2C overexpression accelerated the wound healing capabilities of HepG2 cells (Fig. 3C), whereas KIF2C silencing had the opposite effect on SK-Hep1 cells (Fig. 3D).

In order to test whether KIF2C is required for HCC metastasis *in vivo*, a lung metastasis model was established. According to previous reports (Chang et al., 2012; Li et al., 2019b), we injected one million KIF2C-depleted SK-Hep1, KIF2C-overexpressing HepG2 or the corresponding control cells into nude mice via tail vein (*n* = 8). Two and three nude mice in KIF2C-knockdown SK-Hep1 and the corresponding control groups died from pulmonary embolism by accident, respectively, and no mice in other groups died from pulmonary embolism. Eight weeks post tail vein injections with the indicated cell lines, the mice were sacrificed and the lungs, livers, kidneys and adrenal glands were subjected to Hematoxylin and Eosin (H&E) staining and the tumor nodules were counted. Metastatic tumor nodules were found in lungs only. As shown in Fig. 3E, the volume and number of tumor nodules were decreased in the mice injected with KIF2C-depleted SK-hep1 cell in comparison to the control group. However, the ectopic expression of KIF2C was shown to increase lung metastasis of HepG2 tumors (Fig. 3F). Taken together, these results indicate that KIF2C increases HCC cell migration and invasion *in vitro* and enhances HCC metastasis *in vivo*.

### KIF2C interacts with TBC1D7 in HCC

**Figure 4:**
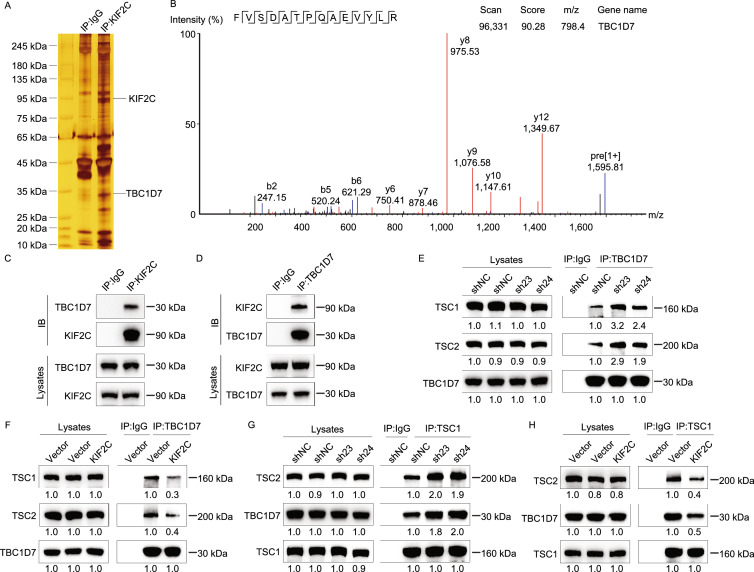
**KIF2C interacts with TBC1D7 and disrupts the TSC complex formation**. (A) Co-immunoprecipitation assay was performed in KIF2C-overexpressing HepG2 cells using normal mouse IgG and KIF2C antibody. The samples were subjected to silver staining to identify potential binding partners of KIF2C protein. (B) Immunoprecipitation samples were digested by trypsin and subjected to mass spectrometry analysis. A unique peptide of TBC1D7 protein was identified in the KIF2C-overexpressing immunoprecipitation samples by analyzing the mass-to-charge ratio of the samples. (C and D) The endogenous interaction of KIF2C and TBC1D7 confirmed by immunoprecipitation assay. Wild type SK-Hep1 cells were harvested for immunoprecipitation and analyzed by Western blot with the indicated antibodies. (E and F) The influence of KIF2C knockdown (E) and overexpression (F) on the TBC1D7-TSC1 and TBC1D7-TSC2 associations was evaluated by immunoprecipitation. (G and H) Immunoprecipitation assay showing that silencing of KIF2C stabilizes the TSC complex (G), but ectopic expression of KIF2C disrupts it (H)

To elucidate the molecular mechanisms underlying the role of KIF2C in promoting HCC progression, an immunoprecipitation assay was performed to detect potential binding partners of KIF2C. Subsequent silver staining and mass spectrometry (MS) analyses identified TBC1D7, a third member of the TSC complex, as a potential binding partner of KIF2C (Fig. 4A and 4B) (Dibble et al., 2012). To validate this finding, epitope-tagged KIF2C and TBC1D7 were overexpressed in HEK293T cells and co-immunoprecipitations were performed, and the results demonstrated a physical association between KIF2C and TBC1D7 (Fig. S3A and S3B). Additionally, endogenous KIF2C was shown to bind to TBC1D7 in HepG2 cells, indicating that KIF2C and TBC1D7 form a complex *in vivo* (Fig. 4C and 4D). Analysis of TCGA data suggested that the expression level of TBC1D7 in HCC was higher than that in normal liver tissues (data not shown). These data strongly support an interaction between KIF2C and TBC1D7 in HCC cells.

### KIF2C disturbs the stability of TSC complex

TBC1D7 is the third subunit of the TSC complex and serves to enhance the stability of the TSC1-TSC2 complex through its direct interaction with TSC1 (Dibble et al., 2012). We proposed that KIF2C-TBC1D7 binding could influence TSC complex formation, and tested this hypothesis by carrying out co-immunoprecipitations. Our results revealed that the interaction of TBC1D7 with TSC1 and TSC2 was enhanced in the SK-Hep1 cells, in which KIF2C was silenced (Fig. 4E). In contrast to this result, when KIF2C was overexpressed in the HepG2 cells, the interaction of TBC1D7 with TSC1 and TSC2 decreased (Fig. 4F). Consistent with these findings, knockdown of KIF2C was shown to promote the interaction of TBC1D7 with TSC1 and TSC2 (Fig. 4G), however, the ectopic expression of KIF2C inhibited these interactions (Fig. 4H). Taken together, these results demonstrate that the interaction between KIF2C and TBC1D7 disrupts the formation of the TSC complex in HCC cells.

### KIF2C exerts an oncogenic function in HCC cells by enhancing mTORC1 signaling

**Figure 5:**
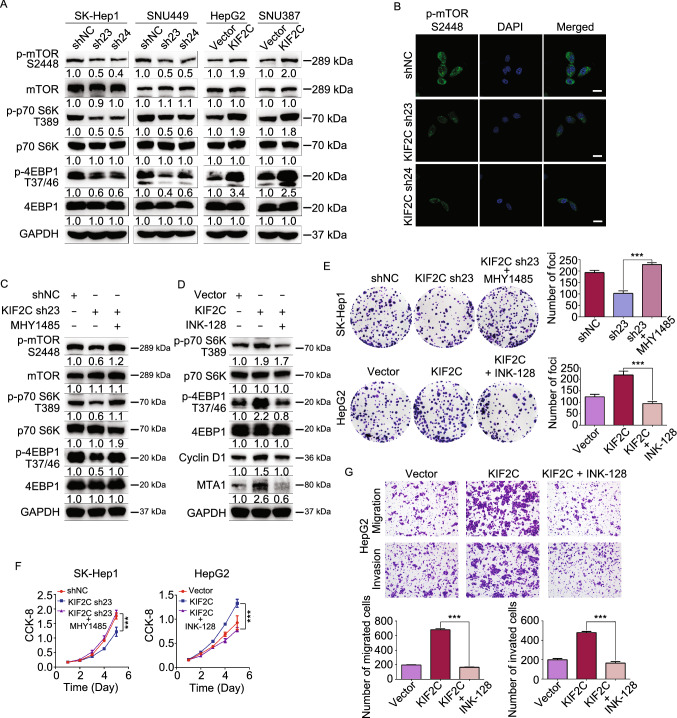
**KIF2C promotes HCC progression by activating mTORC1 signaling**. (A) Western blot analysis of the phosphorylation of mTOR and its substrates (p70 S6K and 4EBP1) in the KIF2C-depleted and -overexpressing HCC cells. (B) Knockdown of KIF2C in SNU449 cell results in a significant decrease of mTOR phosphorylation visualized by immunofluorescence, the cells are counterstained with DAPI to indicate the nucleus. Scale bars, 20 μm. (C) Western blot analysis showing that MHY1485 restores the KIF2C silencing induced phosphorylation inhibition of mTOR, p70 S6K and 4EBP1 in SK-Hep1 cell. (D) Western blot analysis demonstrating that INK-128 treatment eliminates the activity of mTORC1 signaling enhanced by KIF2C overexpression in HepG2 cell. (E) Colony formation assay was used to evaluate the proliferation of the indicated HCC cells. The colonies were captured and counted. Data are presented as the mean ± SD of three independent experiments, ***P < 0.001, Student’s *t*-test. (F) Cellular proliferation of the indicated HCC cells was detected with CCK-8 assay. Data are presented as the mean ± SD of three independent experiments, ***P < 0.001, Student’s *t*-test. (G) Transwell migration and Matrigel invasion assays were employed to determine the migratory and invasive abilities of the indicated HCC cells. The migrated and invaded cells were counted. Data are presented as the mean ± SD of three independent experiments, ***P < 0.001, Student’s *t*-test

Recent studies have shown that TSC2 possesses a GAP domain within its C terminus, and that it acts as a GAP for Rheb, a GTPase that directly controls mTOR activation. TSC complex formation enhances the GAP activity of TSC2, thereby inhibiting mTORC1 signaling (Zhang et al., 2003). The identification of the interaction between KIF2C and TBC1D7, which we show could disrupt the TSC complex, suggests that KIF2C may play a positive role in mTORC1 signal transduction. Results of Western blot analyses revealed that the knockdown of KIF2C reduced the phosphorylation of mTOR, as well as its downstream kinases, p70 S6K, and 4EBP1 (Fig. 5A). However, the overexpression of KIF2C led to an enhancement of the phosphorylation of these kinases (Fig. 5A). Immunofluorescence data also confirmed that KIF2C silencing interfered the mTORC1 signaling in HCC cells (Figs. 5B and S4A). To further verify that KIF2C has an effect on mTORC1 signaling, the KIF2C-depleted SK-Hep1 cells were treated with MHY1485, an agonist of mTORC1 signaling (Choi et al., 2012). Our results indicated that MHY1485 restored the phosphorylation of mTOR, p70 S6K, and 4EBP1 that had initially been decreased as a result of KIF2C silencing in SK-Hep1 cells (Fig. 5C). Furthermore, when the HepG2 cells overexpressing KIF2C were treated with INK-128, an mTOR ATP site inhibitor (Hsieh et al., 2012), the KIF2C-induced increased phosphorylation of p70, S6K, and 4EBP1, as well as enhanced protein levels of Cyclin D1 and MTA1 (Fig. 5D) were eliminated by INK-128, all of which are target genes of mTORC1 signaling (Mamane et al., 2004; Hsieh et al., 2012). These findings demonstrate that KIF2C plays a role in mTORC1 signaling.

Next, we investigated whether mTORC1 signaling plays a role in KIF2C-related HCC progression. As expected, CCK-8 and colony formation assays showed that the inhibitory effect of KIF2C knockdown on HCC cell proliferation was reversed upon the introduction of MHY1485 (Fig. 5E and 5F). Additionally, INK-128 inhibited the enhanced proliferative capacity of HCC cells induced by overexpression of KIF2C (Fig. 5E and 5F). INK-128 has been reported to inhibit the metastasis of prostate cancer cells (Hsieh et al., 2012). Similarly, in the present study, we found that INK-128 treatment inhibited the migration and invasion of HCC cells (Figs. S4B and S4C). Notably, we also observed that INK-128 treatment abolished the augmentation of migration and invasion induced by the overexpression of KIF2C (Fig. 5G and S4D). Overall, these results indicate that KIF2C promotes HCC progression by activating mTORC1 signal transduction.

### KIF2C antagonizes TBC1D7 in mTORC1 signaling and HCC progression

**Figure 6:**
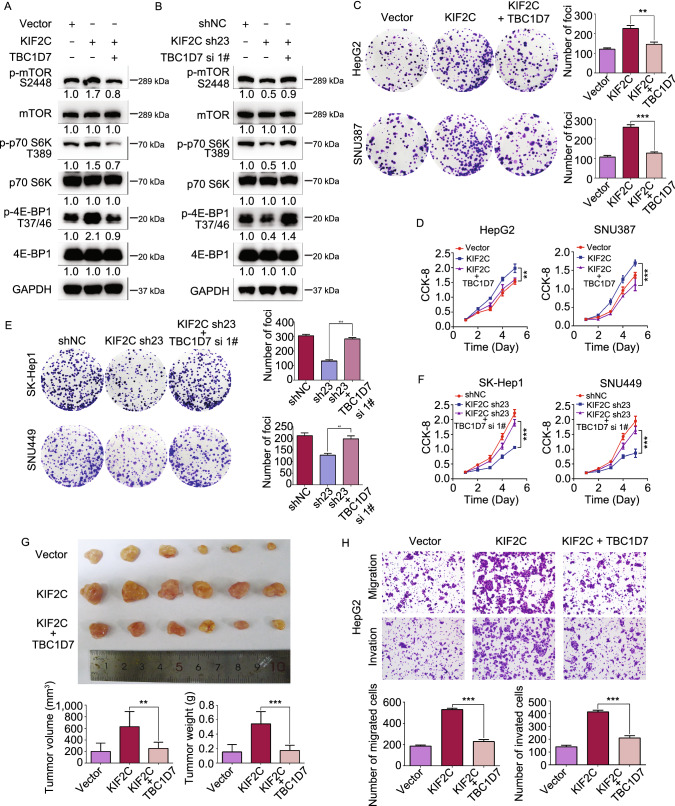
**KIF2C-induced mTORC1 signaling and HCC aggressiveness are antagonized by the overexpression of TBC1D7**. (A) Western blot assay revealing that TBC1D7 overexpression abolishes the KIF2C-induced phosphorylation of mTOR, p70 S6K and 4EBP1 in HepG2 cells. (B) Western blot analyses revealing the effect of KIF2C knockdown or together with TBC1D7 knockdown on the mTOR1 signaling in SK-Hep1 cells. (C and D) Colony formation (C) and CCK-8 (D) assays showing the influence of the KIF2C and TBC1D7 overexpression on HCC cell proliferation. The colonies were captured and counted. Data are presented as the mean ± SD of three independent experiments, **P < 0.01, ***P < 0.001, Student’s *t*-test. (E and F) Colony formation (E) and CCK-8 (F) assays showing the influence of the knockdown of KIF2C or both of KIF2C and TBC1D7 on HCC cell proliferation. Data are presented as the mean ± SD of three independent experiments, **P < 0.01, ***P < 0.001, Student’s *t*-test. (G) TBC1D7 antagonizes the effect of KIF2C on HCC tumor growth. Subcutaneous tumors derived from indicated HepG2 cells were measured by volume and weight. **P < 0.01, ***P < 0.001, Student’s *t*-test. (H) Overexpression of TBC1D7 abolishes the KIF2C-induced migration and invasion of HepG2 cells. The migrated and invaded cells were counted. Data are presented as the mean ± SD of three independent experiments, ***P < 0.001, Student’s *t*-test

The findings that the interaction between KIF2C and TBC1D7 disturbs the formation of the TSC complex, resulting in the enhancement of mTORC1 signaling, led us to propose that KIF2C may antagonize the inhibitory function of TBC1D7 in mTORC1 signaling and HCC progression. To test this hypothesis, TBC1D7 siRNA knockdown and overexpression were performed in KIF2C-delepted and -overexpressed HCC cells, respectively; the efficacies were confirmed by Western blot analysis (Fig. S5A and S5B). TBC1D7 overexpression was observed to abolish the phosphorylation of mTORC1 and its downstream kinases p70 S6K and 4EBP1 that had been induced by the ectopic expression of KIF2C (Fig. 6A). Additionally, knockdown of TBC1D7 reversed the restrained mTORC1 signaling that had been triggered by the silencing of KIF2C in SK-Hep1 cells (Fig. 6B). These results indicated that TBC1D7 antagonizes the activation of mTORC1 signaling induced by KIF2C.

The function of the TBC1D7 and KIF2C interaction in HCC progression was further explored. As expected, colony formation and CCK-8 assays demonstrated that the overexpression of TBC1D7 eliminated KIF2C-enhanced HCC cell proliferation (Fig. 6C and 6D). In concordance with this result, the inhibition of proliferation of HCC cells induced by the silencing of KIF2C was also observed to be relieved by TBC1D7 interference (Fig. 6E and 6F). A xenograft tumor model was established to further explore the role of the TBC1D7 and KIF2C interaction in tumor growth. The ectopic expression of TBC1D7 was shown to reduce KIF2C-increased tumor volume and tumor weight (Fig. 6G). Furthermore, the enhanced migratory and invasive abilities of HCC cells overexpressing KIF2C were inhibited by the overexpression of TBC1D7 (Figs. 6H and S5C). These results indicate that TBC1D7 antagonizes the efficacy of KIF2C in mTORC1 signaling and HCC progression.

### KIF2C is activated in HCC by Wnt/β-catenin signaling

**Figure 7:**
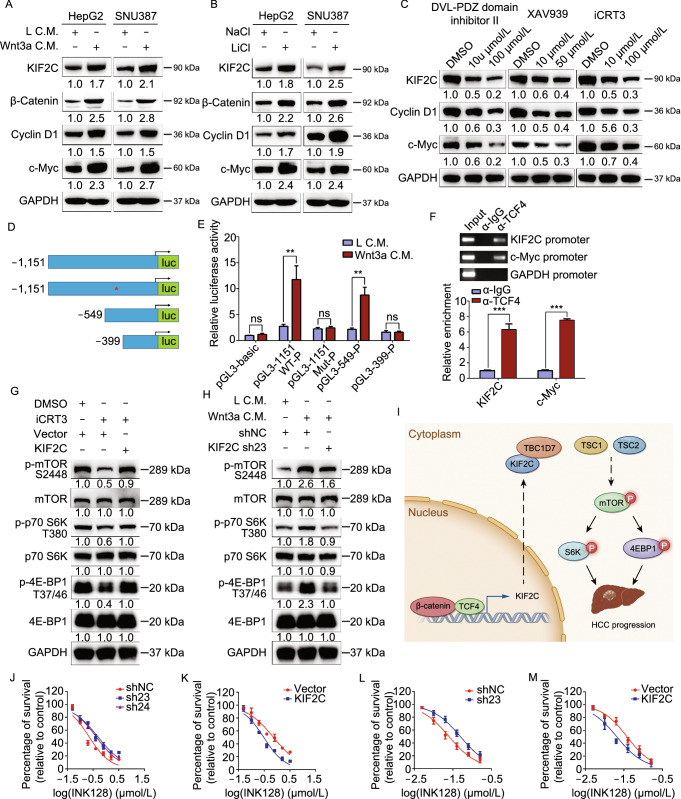
**KIF2C expression is upregulated by Wnt/β-catenin signaling in HCC**. (A) Western blot analyses showing that activation of Wnt/β-catenin by Wnt3a increases the expression level of KIF2C protein in HepG2 and SNU387 cells. (B) The effect of LiCl treatment on the expression of KIF2C in HepG2 and SNU387 cells as examined by Western blot. (C) Western blot analysis of the expression level of KIF2C protein in SK-Hep1 cells treated with Wnt antagonists (including Dvl-PDZ domain inhibitor II, XAV939 and iCRT3). (D) Schematic illustration of the luciferase reporters driven by the KIF2C promoter and its mutants. (E) Dual-luciferase assay in 293T cells suggests that the putative TCF4 binding site is essential for the responsiveness of KIF2C promoter to Wnt3a treatment. Data are presented as mean ± SD of three independent experiments, ns, non-significant, **P < 0.01, Student’s *t*-test. (F) ChIP assay performed in SK-Hep1 cells with anti-TCF4 antibody or control IgG. c-Myc acts as a positive control. Data are presented as mean ± SD of three independent experiments, ***P < 0.001, Student’s *t*-test. (G) Western blot assay revealing that overexpression of KIF2C restores iCRT3-inhibited phosphorylation of mTOR1, p70 S6K and 4EBP1in HepG2 cells. (H) Wnt3a-enhanced mTORC1 signaling is dismissed by knockdown of KIF2C in SK-Hep1 cells. (I) The proposed model of the function of KIF2C in the activation of mTORC1 signaling during HCC pathogenesis. KIF2C is transcriptionally activated by Wnt/β-catenin signaling, then interacts with TBC1D7 to disrupt the stability of the TSC complex, thereby enhancing mTORC1 signaling and HCC progression. (J and K) Cell viability assay (CCK-8 assay) shows the effect of INK128 on KIF2C-depleted SK-Hep1 (J) and KIF2C-overexpressing HepG2 (K) cells. Cells were treated with INK128 for 72 h and measured by CCK-8. (L and M) The effect of INK128 on KIF2C-depleted SK-Hep1 (L) and KIF2C-overexpressing HepG2 (M) cells was detected by colony formation assay

The regulation of KIF2C expression in HCC was investigated by analyzing the KIF2C promoter (≈1.5 kb region) using the PROMO software to identify potential transcription factor binding sites. A potential TCF4 binding site (5′-CTTTGA-3′) was identified in this region, suggesting that Wnt signaling may drive KIF2C expression (Fig. S6A). To test this finding, the Wnt/β-catenin pathway was stimulated using Wnt3a in HCC cells and subsequent qRT-PCR and Western blot analyses were carried out, which demonstrated that both the RNA and protein levels of KIF2C were increased after Wnt activation (Figs. S6B and 7A). In accordance with this finding, treating HCC cells with Lithium chloride (LiCl), which acts as an agonist of the canonical Wnt signaling by inhibiting GSK-3β (Hedgepeth et al., 1997), was shown to enhance the KIF2C expression (Figs. S6C and 7B). To further explore the effect of the Wnt signaling pathway on KIF2C expression, we inhibited the Wnt/β-catenin pathway using three Wnt antagonists, Dvl-PDZ domain inhibitor II (which disrupts the interaction of Dvl2 and LRP6 by targeting the PDZ domain of Dvl2) (Grandy et al., 2009), XAV939 (which stabilizes Axin by blocking tankyrase activity), and iCRT3 (which restrains the transcriptional activity of TCF4 by disrupting the β-catenin/TCF4 association) (Huang et al., 2009; Gonsalves et al., 2011). The results showed that inhibiting the Wnt/β-catenin signaling pathway resulted in decreased KIF2C expression (Figs. 7C and S6D–F).

To further investigate the transcriptional regulation of KIF2C by TCF4, the putative 1151-bp KIF2C promoter region, containing the predicted TCF4 binding site, was cloned into the pGL3-basic luciferase reporter plasmid (Fig. 7D). Additional luciferase reporter plasmids containing mutated (5′-CTTTGA-3′ to 5′-AGGGTC-3′) and truncated promoters were also generated and used as controls (Fig. 7D). Wnt3a treatment was shown to strongly promote the transcriptional activity of 1151-WT-P, suggesting the 1151-bp KIF2C promoter region is regulated by canonical Wnt signaling (Fig. 7E). The reporter plasmids containing either the mutated or truncated (399-P) promoter, which no longer contain the TCF4 binding site, exhibited a greatly reduced responsiveness to Wnt3a treatment compared to the wild type promoter and the truncated promoter (549-P) that retain the TCF4 binding site (Fig. 7E). These results support the hypothesis that the predicted TCF4 binding site identified in the KIF2C promoter region is critical for the transcriptional regulation of KIF2C mediated by the Wnt signaling pathway. Chromatin immunoprecipitation (ChIP) assay confirmed the binding of endogenous TCF4 to the KIF2C promoter (Fig. 7F), indicating that KIF2C is a direct target of TCF4 in HCC cells. In addition, correlation (r) analysis showed that KIF2C expression was highly correlated with Wnt-regulated gene expression (Fig. S6G) (Chen et al., 2016), highlighting the concordance of Wnt/β-catenin activity and KIF2C expression in HCC.

The results of our study thus far, including the identification of KIF2C as a direct target gene of the Wnt/β-catenin signaling pathway and the essential role KIF2C plays in mTORC1 signal transduction, led us to propose that KIF2C may play a pivotal role in the crosstalk between canonical Wnt signaling and mTORC1 signaling. To test this hypothesis, we performed Western blot assay and discovered that the inhibition of canonical Wnt signaling by iCRT3 evidently resulted in the suppression of mTORC1 signal transduction, and the overexpression of KIF2C relieved this suppression (Fig. 7G). Furthermore, the silencing of KIF2C abolished the augmentation of the mTORC1 signaling induced by Wnt3a stimulation (Fig. 7H). Together, these results revealed that KIF2C plays a crucial role in mediating the crosstalk between canonical Wnt signaling and mTORC1 signaling.

Taken together, our results indicate that upon transcriptional activation induced by Wnt/β-catenin signaling, KIF2C binds to TBC1D7, and the KIF2C/TBC1D7 interaction results in the dissociation of the TSC complex, thereby promoting mTORC1 signal transduction and HCC progression (Fig. 7I).

To test the potential of KIF2C as a therapeutic target in the treatment of HCC, we evaluated the impact of silencing or overexpressing KIF2C on the efficacy of the mTOR inhibitor INK128. CCK-8 assay results showed that the IC_50_ of INK128 was obviously increased in KIF2C-depleted SK-Hep1 cells as compared to the control cells (Fig. 7J). The ectopic expression of KIF2C was shown to reduce the IC_50_ of INK128 in HepG2 cells (Fig. 7K). The results of the colony-formation assay further confirmed these findings (Figs. 7L, 7M, S6H and S6I). These results indicated that HCC cells with high expression levels of KIF2C were more sensitive to the mTOR inhibitor INK128.

## Discussion

In this study, we provide experimental evidence showing that KIF2C is upregulated in HCC tissues and that KIF2C up-regulation is associated with a more aggressive malignancy and a poor prognosis. Furthermore, our data support a crucial role for KIF2C in the proliferation and metastasis of HCC. We showed that KIF2C strengthened mTORC1 signaling through its interaction with TBC1D7, and we also provide experimental evidence showing that this interaction results in the disassociation of the TSC complex. Interestingly, KIF2C up-regulation was found to be directly induced by Wnt/β-catenin signaling, revealing a crosstalk between canonical Wnt signaling and mTORC1 signaling. Our findings provide a better understanding of the interaction between Wnt/β-catenin and mTORC1 signaling in the pathogenesis of HCC.

The data from the TCGA and Oncomine databases in addition to our cohort clearly show that KIF2C is frequently up-regulated in human HCC tissues. Consistent with our results, previous studies have reported the overexpression of KIF2C in several cancer types (Nakamura et al., 2007; Ishikawa et al., 2008; Shimo et al., 2008; Bie et al., 2012; Gan et al., 2019). Further analysis revealed that high levels of KIF2C expression were significantly associated with tumor differentiation, recurrence, and a poorer prognosis in HCC patients, suggesting that the upregulated expression of KIF2C in HCC may play a role in malignancy. To test this hypothesis, we carried out a series of *in vivo* and *in vitro* assays to investigate the roles of KIF2C in the regulation of HCC growth, invasion, and metastasis. We found that the ectopic overexpression of KIF2C substantially promoted HCC cell growth, invasion, and migration. In contrast, KIF2C knockdown in HCC cells was shown to inhibit both proliferation and metastasis. These results indicate that KIF2C plays a crucial role in HCC aggressiveness and may exert an oncogenic function in the pathogenesis of HCC. Consistent with our findings, Zhang et al. recently revealed that KIF2C aggravates the HCC progression (Zhang et al., 2020), which highlights the credibility of our data. In addition to KIF2C, several other kinesin superfamily proteins have also been found to be highly expressed in HCC and promote the progression of HCC (Chen et al., 2017; Huang et al., 2018; Luo et al., 2018; Li et al., 2019a; Teng et al., 2019; Li et al., 2020), indicating that kinesin superfamily proteins may be promising prognostic biomarkers and therapeutic targets for HCC.

KIF2C is an M-kinesin, belonging to kinesin 13 family, and contains a motor domain in the middle of its amino acid sequence. KIF2C has been suggested to play a role in the regulation of microtubule depolymerization, spindle assembly, chromosome congression and segregation, kinetochore-microtubule attachment, and cytoskeletal remodeling (Moore et al., 2005; Lee et al., 2008; Honnappa et al., 2009; Tanenbaum et al., 2011; Braun et al., 2014; Eichenlaub-Ritter, 2015; Ritter et al., 2015). In this study, we identified TBC1D7 as binding partner of KIF2C, and we showed further evidence to support that this interaction promotes HCC growth, invasion, and metastasis. TBC1D7 is the third subunit of the TSC complex, and has been reported to bind and stabilize the TSC1/TSC2 complex, and also serves as a suppressor of mTORC1 signaling (Dibble et al., 2012; Capo-Chichi et al., 2013). We showed that the KIF2C/TBC1D7 interaction disrupts the formation of the TSC complex, resulting in the activation of mTORC1 signaling during HCC progression. Our findings provide further evidence for the interaction of KIF2C with TBC1D7, and demonstrated that this interaction is critical for TSC complex disassociation, implying that this interaction is very important in the activation of mTORC1 signaling and HCC progression.

mTORC1 signaling controls cell growth, proliferation, and metabolism. Increased mTORC1 signaling activity is involved in the development of a variety of human cancers (Inoki et al., 2005; Saxton and Sabatini, 2017). mTORC1 signaling activity is shown to be upregulated in 40%–50% of HCCs and has been shown to play a pivotal role in the development and progression of HCC (Villanueva et al., 2008; Bhat et al., 2013; Matter et al., 2014). Our studies demonstrated that KIF2C is an essential positive regulator of mTORC1 signaling in HCC. To test the role of the KIF2C in mTORC1 signaling and HCC progression, we treated KIF2C-overexpressing and KIF2C-depleted HCC cells with the mTOR agonist MHY1485 and the mTOR inhibitor INK-128, respectively. We found that MHY1485 restored the decreased proliferation of HCC cells caused by KIF2C silencing, whereas INK128 reduced the enhanced proliferation of HCC cells induced by KIF2C overexpression. Thus, mTORC1 signaling mediates the enhanced proliferation of HCC cells induced by KIF2C. Beyond its significance in controlling cell proliferation and metabolism, mTORC1 signaling is also essential for tumor cell migration and invasion (Gulhati et al., 2011; Hsieh et al., 2012; Guo et al., 2018). Our results confirmed this assertion on account of INK-128 treatment inhibiting the migration and invasion of HCC cells. Furthermore, we observed that HCC cells with higher levels of KIF2C are more sensitive to the mTOR inhibitor INK128 as compared to the control cells. These results indicate that an mTOR inhibitor may be a potential therapeutic reagent for HCC that is shown to have high expression levels of KIF2C.

The Wnt/β-catenin pathway is a critical contributor to HCC progression, and according to previous studies, alterations in Wnt signaling are identified in 44% of HCCs (Clevers and Nusse, 2012; Ally et al., 2017). Nuclear accumulation of the β-catenin protein, a hallmark of the activation of canonical Wnt pathway, has been identified in 40%–70% of HCCs (Lachenmayer et al., 2012; Pez et al., 2013). Many Wnt/β-catenin targets, such as Cyclin D1 and c-Myc, are essential for the growth and metastasis of HCC (Khalaf et al., 2018). The results of our study demonstrated that KIF2C is a direct target of Wnt/β-catenin signaling, which provides an explanation for the frequent overexpression of KIF2C observed in HCC. Our results suggest that the functions of KIF2C in HCC proliferation and metastasis may be partly due to its activation by Wnt/β-catenin signaling.

The crosstalk between different signaling pathways is crucial for tumorigenesis. Wnt has been reported to activate mTORC1 signaling by repressing the GSK3-dependent phosphorylation of TSC2 (Inoki et al., 2006). Our findings confirmed the activation of mTORC1 signaling by canonical Wnt signaling. Furthermore, it is interesting that the inhibition of TCF4 transcriptional activity by iCRT3 treatment also reduced mTORC1 signal transduction in HCC cells. This finding implies a GSK3-independent manner of mTORC1 signaling activation mediated by the Wnt pathway. Taken together, our study indicates that KIF2C plays a crucial role as a mediator of the crosstalk between Wnt/β-catenin signaling and mTORC1 signaling during the progression of HCC.

In summary, in the present study, we showed that KIF2C is highly expressed in HCC and that this high expression is correlated with a more aggressive malignancy. We demonstrated that KIF2C is activated by the Wnt/β-catenin signaling pathway and plays a crucial role in mTORC1 signaling and HCC progression through its interaction with TBC1D7, which results in the disruption of the TSC complex. Considering the significance of the Wnt/β-catenin-KIF2C-mTORC1 axis in HCC progression, our study highlights the potential of KIF2C as a promising therapeutic target for the treatment of HCC.

## Materials and methods

### Patients and specimens

qPCR and Western blot analyses of KIF2C expression were carried out on 39 and 12 paired HCC and adjacent tissues, respectively. All these tissues were obtained from the Biological Specimen Bank of Sun Yat-sen University Cancer Center (SYSUCC), Guangzhou, China. Paraffin-embedded pathological specimens from 149 patients with HCC were collected from the archives of the Department of Pathology in our institute, between January 2013 and November 2015, and used for immunohistochemical analysis. The cases were selected based on having a distinctive pathological diagnosis of HCC, undergoing primary and curative resection for HCC, availability of resection tissue, complete follow-up data, and had not received preoperative anticancer treatment. Written informed consent was obtained from all patients prior to their participation in the study. All of the samples used in this study were approved by the Committees for Ethical Review of Research Involving Human Subjects in the Sun Yat-sen University Cancer Center.

### *In vivo* experiments

Five-week-old male BALB/c nude mice were used in this study. For the subcutaneous xenograft model, the control and experimental HCC cells (2 × 10^6^) were suspended in 100 μL PBS and then injected subcutaneously into the flanks of the nude mice. Tumor growth was examined over the course of four weeks, and after four weeks, the mice were sacrificed and the tumors were harvested, fixed and paraffin-embedded for further analysis. For the metastatic assay, the control and experimental HCC cells (1 × 10^6^) were injected into the tail vein of each mouse. After 8 weeks, the mice were euthanized, examined, and the lungs were excised and embedded in paraffin for further analysis. The animal studies were approved by the SYSUCC Institutional Animal Care and Usage Committee and performed in accordance with the SYSUCC animal care guidelines.

### Cell culture

LO2, Hep3B, and Huh7 are purchased from ATCC. SNU449 and SNU387 are kindly gifted by Professor Xiaofeng Zheng (Sun Yat-sen University Cancer Center), SK-Hep1 and HepG2 are gifts from professor Yunfei Yuan (Sun Yat-sen University Cancer Center). All cell lines were authenticated. All of the cells were cultured in Dulbecco’s modified Eagle’s medium (DMEM) supplemented with 10% FBS and 1% Penicillin-Streptomycin in a 37 °C humidified incubator with a 5% CO_2_ environment.

### Plasmids construction and transfection

The coding sequences (CDS) of human KIF2C and TBC1D7 were amplified and cloned into the pLVX-myc-mcs-3xflag lentivirus vector and pLV-CMV-MCS-3Xflag-IRES-Bla, respectively, and used for stable overexpression experiments. Transient overexpression experiments were carried out by amplifying the coding sequences (CDS) of TBC1D7 and cloning it into the pCS2-HA vector. For the knockdown assay, two short KIF2C shRNA (sh23: 5′-GCATAAGCTCCTGTGAATATA-3′ and sh24: 5′-GCAGGCTAGCAGACAAATAAG-3′), and the scramble control shRNA (shNC: 5′-GCTTCGCGCCGTAGTCTTA-3′) lentiviral vector were purchased from the GeneCopoeia Company. Two short TBC1D7 siRNAs (siRNA 1#: 5′-GCGGGATGTTTGCCTGAAT-3′ and siRNA 2#: 5′-GAAAGTCGTTCGCTTTGTT-3′) and a control siRNA were purchased from the RIBOBIO Company. Cell transfection was performed using Lipofectamine 2000 (11668019, Invitrogen) following the manufacturer’s instructions.

### Cell proliferation, colony formation, migration and invasion assay

The indicated HCC cells were plated in 96-well plates at a density of 1000 cells/well, and proliferation was detected using the CCK-8 Kit (HY-K0301, MCE) according to the manufacture’s instruction. For the colony formation assay, 500 cells were seeded in a 6-well plate and maintained with 10% FBS DMEM for 2 weeks. Colonies were fixed with methanol for 30 min and subsequently stained with 0.1% crystal violet for 1 h. Clones with more than 50 cells were defined as positive. The Transwell migration and Matrigel invasion assays were performed as previously described (Teng et al., 2019). Briefly, we put the chamber into the 24-well plate (filled with 600 μL 10% FBS-DMEM/well), then seeded 5 × 10^4^ HCC cells (suspended with 300 μL serum-free DMEM) to the upper chamber. For Transwell migration, after incubation for 10 h (SK-Hep1 and SNU449 cells) or 24 h (HepG2 and SNU387 cells), the migrated cells were fixed with methanol and stained with crystal violet. For Matrigel invasion, after incubation for 24 h (SK-Hep1 and SNU449 cells) or 36 h (HepG2 and SNU387 cells), the migrated cells were fixed with methanol and stained with crystal violet. The migrated and invaded cells were captured and calculated using microscope.

### Immunoprecipitation and Western blot analyses

For immunoprecipitation assay, the indicated cells were harvested and lysed with TNE lysis buffer (10 mmol/L Tris-HCl, pH 7.5, 150 mmol/L NaCl, 2 mmol/L EDTA, and 0.5% Nonidet P-40) containing a protease inhibitor cocktail. Lysates were subjected to immunoprecipitation with the indicated antibodies followed by an incubation period with protein A/G beads at 4 °C for 4 h. The precipitates were then washed and boiled in order to isolate the bound proteins. The samples were then analyzed using Western blot, silver staining, and mass spectrometry (MS). Silver staining was carried out using the Fast Silver Stain Kit by following the manufacturer’s instructions (P0017S, Beyotime) and MS was performed by Wininnovate Bio, in Shenzhen, China.

Western blot analysis was carried out by first electrophoresing the proteins through an SDS-PAGE gel and subsequently transferring them to 0.22 μm PVDF membrane (Roche). The membrane was blocked with 5% milk for 30 min, and then incubated with the indicated primary antibody overnight at 4 °C. After three washes with TBST (each for 5 min), the membrane was incubated with the appropriate secondary antibodies at room temperature for 1 h, washed with TBST, and visualized using the ECL by ChemiDoc™ Touch Imaging System (Bio-Rad).

### Immunofluorescence analysis

The cells were cultured in glass dishes, fixed in 4% paraformaldehyde/PBS at room temperature for 30 min, and then permeabilized using 0.2% Triton X-100/PBS for 15 min. After a blockage in 1.5% FBS/PBS for 30 min, the cells were incubated with the indicated primary antibodies overnight at 4 °C. After three washes with PBS, the cells were incubated with appropriate fluorescent secondary antibodies, and then 4’,6-Diamidine-2’-phenylindole dihydrochloride (DAPI, C1002, Beyotime) for staining of nuclei. The immunofluorescence images were then captured using a confocal microscope (Olympus FV1000, Japan).

### Antibodies

The antibodies used in this study are as follows. KIF2C: sc-81305, Santa Cruz; TBC1D7: #14949, Cell Signaling Technology; TSC1: #6935, Cell Signaling Technology; TSC2: #4308, Cell Signaling Technology; mTOR: #2983, Cell Signaling Technology; p-mTOR: #5536, Cell Signaling Technology; p70 S6K: #2780, Cell Signaling Technology; p-p70 S6K: #9234, Cell Signaling Technology; 4EBP1: #9644, Cell Signaling Technology; p-4EBP1: #2855, Cell Signaling Technology; β-catenin: #8480, Cell Signaling Technology; cyclin D1: #2922, Cell Signaling Technology; c-Myc: A1309, Abclonal; GAPDH: 60004-1-Ig, Proteintech.

### Quantitative RT-PCR

Total RNA was extracted with Trizol (15596026, Invitrogen) and cDNA was synthesized using the PrimeScript RT-PCR kit (RR055A, Takara) according to the manufacturer’s instructions. A SYBR Green PCR Kit (1725125, Bio-Rad) was used to conduct qRTPCR. Primer sequences are as follows: qRT-KIF2C (F: 5′-GGAGGAGAAGGCTATGGAAGA-3′, R: 5′-TCGCAGGGCTGAGAAATG-3′); qRT-cyclin D1 (F: 5′-ATCAAGTGTGACCCGGACTG-3′, R: 5′-CTTGGGGTCCATGTTCTGCT-3′); qRT-c-Myc (F: 5′-TACAACACCCGAGCAAGGAC-3′, R: 5′-GAGGCTGCTGGTTTTCCACT-3′); qRT-GAPDH (F: 5′-TGCACCACCAACTGCTTAGC-3′, R: 5′-GGCATGGACTGTGGTCATGAG-3′).

### Dual-luciferase reporter assay

The KIF2C promoter and its variants were cloned into the pGL3-Basic plasmid, which were then transfected into HepG2 cells. The Renilla luciferase reporter was used as an internal control. 24 h post-transfection, the HepG2 cells were stimulated overnight with either L conditioned medium (L C.M.) or Wnt3a conditioned medium (Wnt3a C.M.) and then harvested for luciferase activity assay. Each luciferase reporter assay was conducted in triplicate, and the average activity was calculated.

### Chromatin immunoprecipitation (ChIP)

ChIP assay was performed according to the manufacturer’s instruction using the ChIP kit (17-371, Millipore). In brief, cell lysates were sonicated to shear DNA to 200–500 bp fragments and were then immunoprecipitated using the TCF4 antibody (Abcam, ab4078, 1:200, UK). Input and immunoprecipitated DNA was then subjected to PCR analysis using primers specific to the regulated gene promoters. The ChIP-primers used in this study are as follows: ChIP-KIF2C (F: 5′-GGTGGTAGACTTCACTCAATAA-3′, R: 5′-CGAGATCAGAAGTTCGAGAC-3′); ChIP-c-Myc (F: 5′-CAGAGAAAGGGAGAGGGTTTG-3′, R: 5′-GAGCACTCTAGCTCTAGGATGTA-3′); ChIP-GAPDH (F: 5′-TACTAGCGGTTTTACGGGCG-3′, R: 5′-TCGAACAGGAGGAGCAGAGAGCGA-3′).

### Statistical analysis

Statistical analysis was performed using the SPSS 20.0 software (SPSS, Chicago, IL, USA). Receiver operating characteristic (ROC) curve analysis was applied to determine the cut-off score for the high expression of KIF2C (Zhu et al., 2012). Associations between KIF2C expression and the clinicopathological features were analysed using the Pearson chi-square test. The statistical significance of the correlation between KIF2C expression and disease-specific survival was estimated using the log-rank test. Multivariate survival analyses were performed using the Cox proportional hazards regression model. The statistical significance between two groups was compared with the two-tailed Student’s *t*-test. Data are presented as mean ± SD and a *P* value of less than 0.05 was considered to be statistically significant.

## Electronic supplementary material

Below is the link to the electronic supplementary material.

Supplementary material 1 (PDF 1048 kb)
