# Supplementary material for: KIF2C: a novel link between Wnt/β-catenin and mTORC1 signaling in the pathogenesis of hepatocellular carcinoma
Source: Protein Cell. 2020 Aug 3;12(10):788–809. doi: 10.1007/s13238-020-00766-y (PMC8464548; doi:10.1007/s13238-020-00766-y)
Supplement: Supplementary file 1 — Supplementary material 1 (PDF 1048 kb) [file 13238_2020_766_MOESM1_ESM.pdf]

## SUPPLEMENTARY MATERIALS

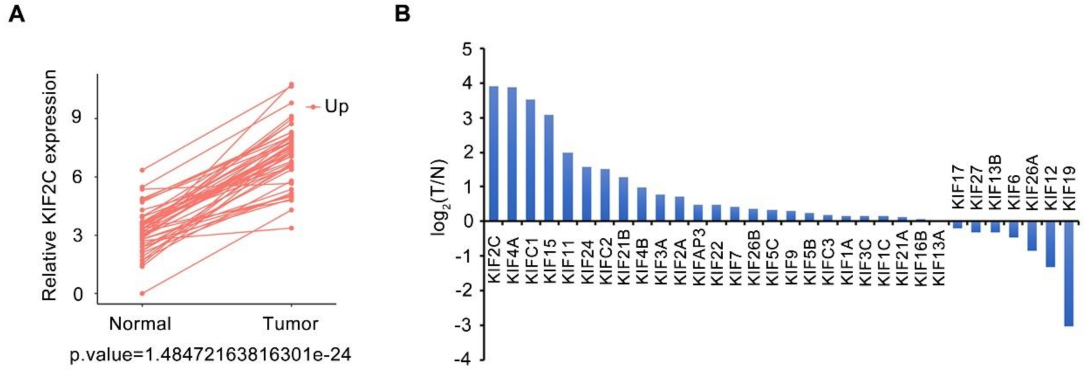


**Figure S1. KIF2C expression is upregulated in HCC tissues.** (A) Expression levels of KIF2C in the paired HCC and adjacent liver tissues derived from the TCGA database. (B) The expression of kinesin families in HCC derived from the TCGA database.

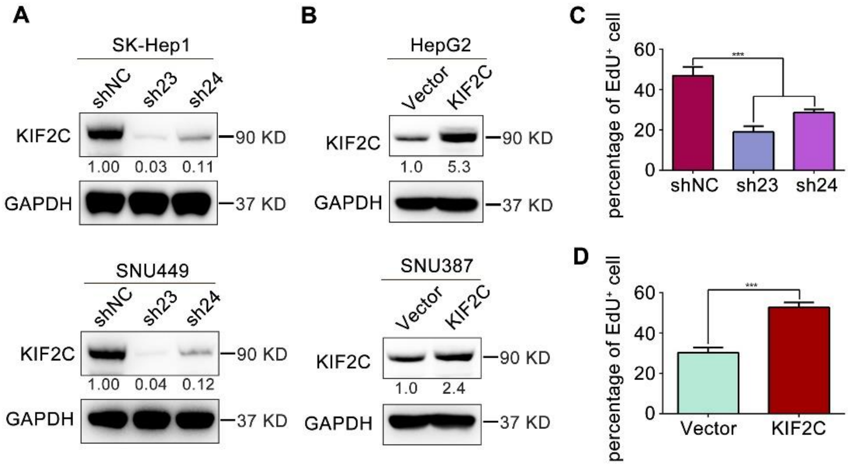


**Figure S2. KIF2C promotes HCC cell proliferation.** (A) The knockdown efficiencies of KIF2C small hairpin RNAs (shRNAs) were confirmed by western blot in SK-Hep1 and SNU449 cells. (B) Western blot analyses confirm the ectopic expression of KIF2C in the stably transfected HepG2 and SNU387 cells. (C and D). The percentages of EdU+ cells were calculated in KIF2C-depleted SK-Hep1 (C) and KIF2C-overexpressed HepG2 (D) cells. Data are presented as the mean ± SD of three independent experiments, ** $P < 0.01$, *** $P < 0.001$, Student's t-test.

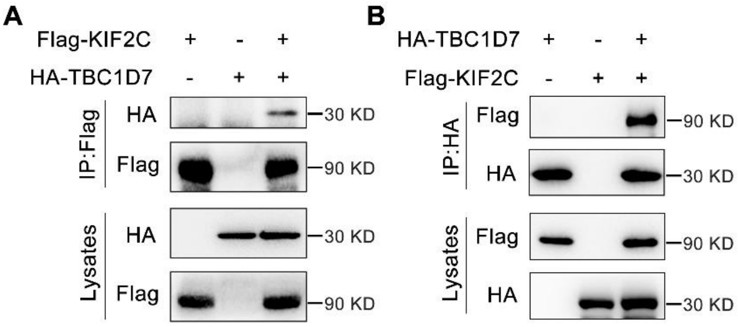


**Figure S3. KIF2C interacts with TBC1D7.** (A and B) Immunoprecipitation shows that an association of KIF2C and TBC1D7 is observed in the system of ectopic overexpression KIF2C and/or TBC1D7. HEK293T cells were transfected with expression plasmids encoding Flag-tagged KIF2C and HA-tagged TBC1D7 for 48 hours, then harvested for immunoprecipitation and examined by western blot with the indicated antibodies.

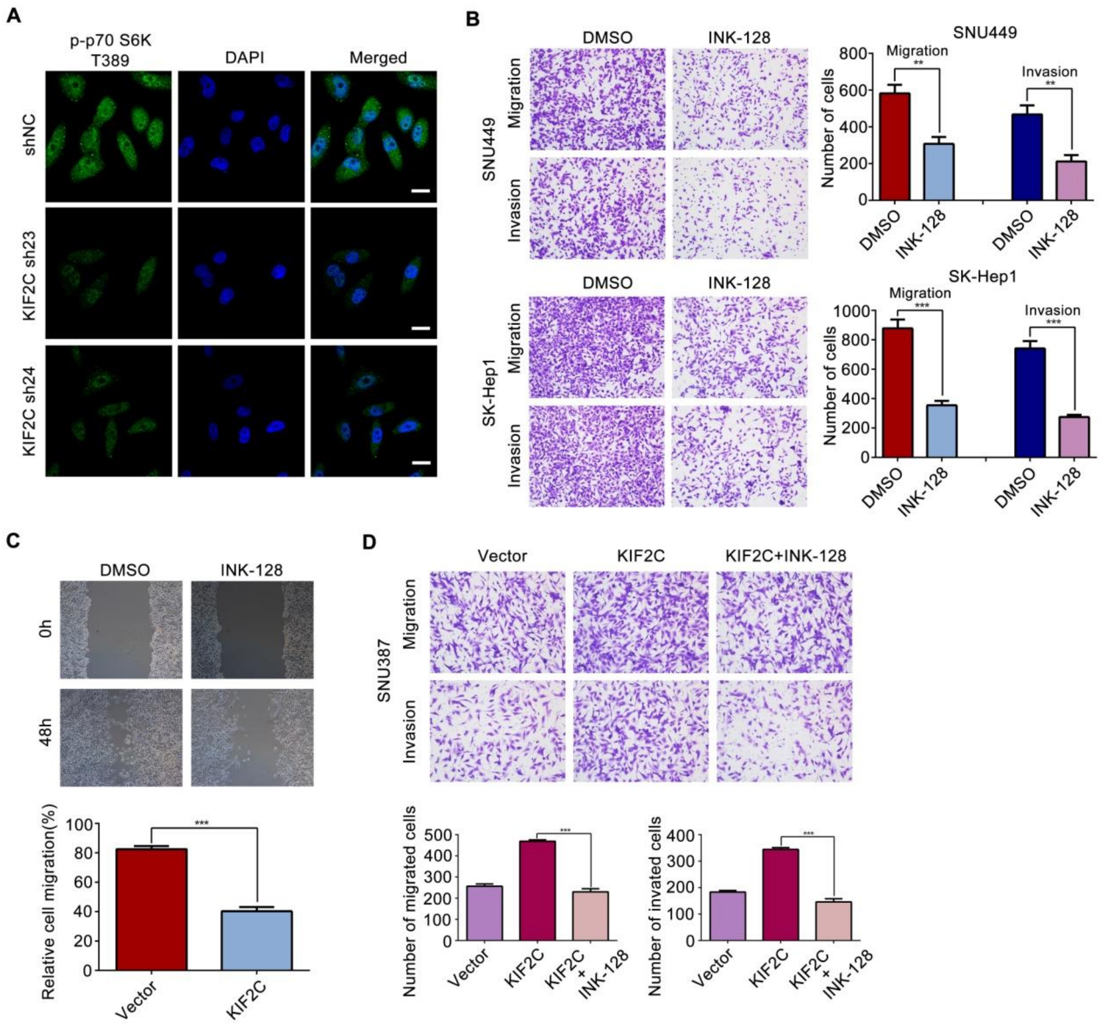


**Figure S4. KIF2C promotes HCC progression through mTOR signaling.** (A) The expression of phosphorylated p70 S6K in KIF2C-silencing or control SNU449 cells is visualized by immunofluorescence. The cell was counterstained with DAPI to indicate the nucleus. Scale bars, 20 μm. (B) Treatment of INK-128 inhibits the migratory and invasive abilities of HCC cells. The migrated and invaded cells were counted. Data are presented as the mean ± SD of three independent experiments, ** P < 0.01, *** P < 0.001, Student's t-test. (C) Wound healing assay reveals that INK-128 treatment inhibits SK-Hep1 cell migration. Data are presented as the mean ± SD of three independent experiments, *** P < 0.001, Student's t-test. (D) Treatment of INK-128 eliminates KIF2C-promoted migration and invasion of SNU387 cells. Data are presented as the mean ± SD of three independent experiments, *** P < 0.001, Student's t-test.

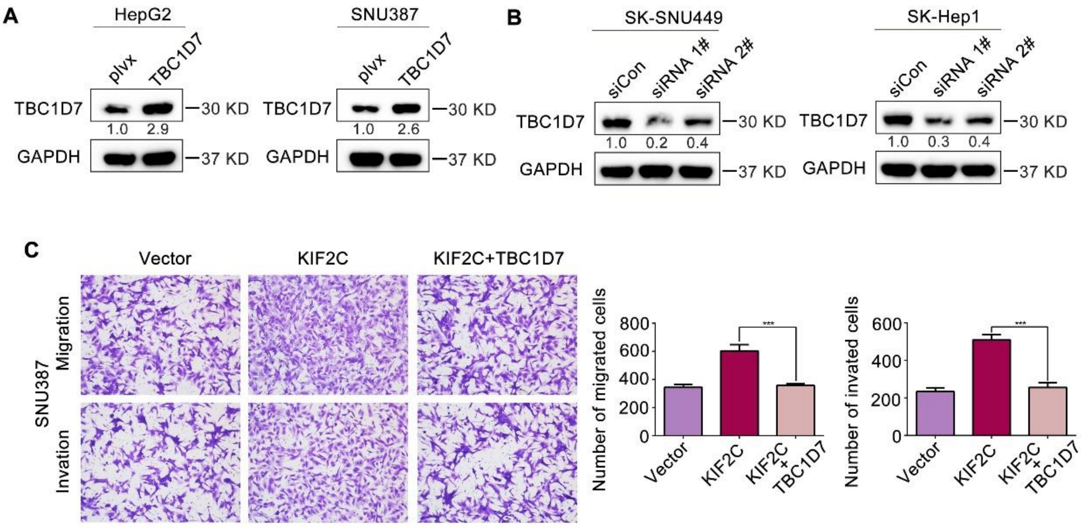


**Figure S5. KIF2C-enhanced HCC aggressive phenotypes are antagonized by TBC1D7 overexpression**

(A) Western blot analyses confirm the ectopic expression of TBC1D7 in transient transfected HepG2 and SNU387 cells. (B) The knockdown efficiency of TBC1D7 siRNAs is confirmed by western blot analysis in SK-Hep1 and SNU449 cells. (C) Overexpression of TBC1D7 eliminates KIF2C-induced migratory and invasive abilities of SNU387 cells. The migrated and invaded cells were counted. Data are presented as the mean ± SD of three independent experiments, *** $P < 0.001$, Student’s t-test.

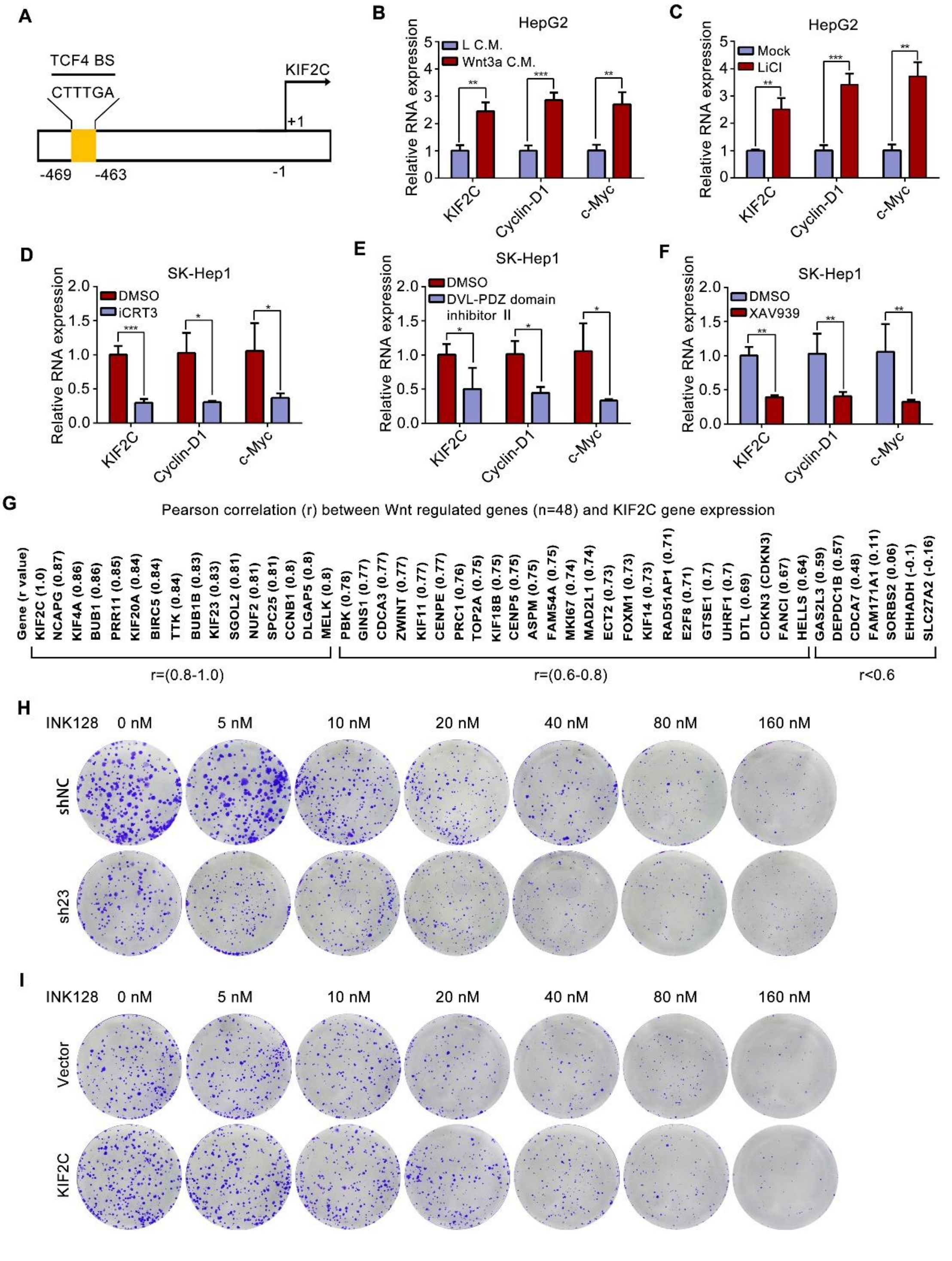


**Figure S6. KIF2C is activated by Wnt/β-catenin signal pathway.** (A) A TCF4-binding site (TCF4 BS) is identified in the KIF2C promoter using the PROMO software. (B) HepG2 cells were treated with Wnt3a, and then qRT-PCR assays were utilized to examine the mRNA expression of KIF2C, CyclinD1, c-Myc and the

Wnt/β-catenin target genes. Data are presented as mean ± SD of three independent experiments, ** $P < 0.01$, *** $P < 0.001$, Student's t-test. (C) The effect of LiCl treatment on the expression of KIF2C is determined by qRT-PCR assays. Data are presented as mean ± SD of three independent experiments, ** $P < 0.01$, *** $P < 0.001$, Student's t-test. (D) Inhibition of Wnt/β-catenin by iCRT3 decreases the mRNA expression of KIF2C. Data are presented as mean ± SD of three independent experiments, * $P < 0.05$, *** $P < 0.001$, Student's t-test. (E) Treatment of Dvl-PDZ domain inhibitor II reduces the mRNA expression of KIF2C in SK-Hep1. Data are presented as mean ± SD of three independent experiments, * $P < 0.05$, Student's t-test. (F) The mRNA expression of KIF2C is decreased by the treatment of XAV939. Data are presented as mean ± SD of three independent experiments, * $P < 0.05$, *** $P < 0.001$, Student's t-test. (G) Pearson correlation (r) analysis of KIF2C and 48 Wnt-regulated genes expression in TCGA database revealed that KIF2C expression was highly correlated with Wnt-regulated genes expression. (H and I) Representative images of the colony formation of KIF2C-depleted SK-Hep1 (H) and KIF2C-overexpressed HepG2 (I) cells treated with increasing concentration of INK128 for two weeks.
